# EEG theta and Mu oscillations during perception of human and robot actions

**DOI:** 10.3389/fnbot.2013.00019

**Published:** 2013-11-13

**Authors:** Burcu A. Urgen, Markus Plank, Hiroshi Ishiguro, Howard Poizner, Ayse P. Saygin

**Affiliations:** ^1^Department of Cognitive Science, University of California San DiegoSan Diego, CA, USA; ^2^Qualcomm Institute, California Institute of Telecommunications and Information Technology, University of California San DiegoSan Diego, CA, USA; ^3^Institute for Neural Computation, University of California San DiegoSan Diego, CA, USA; ^4^Osaka UniversityOsaka, Japan; ^5^Advanced Telecommunications ResearchKeihanna Science City, Japan; ^6^Neurosciences Program, University of California San DiegoSan Diego, CA, USA

**Keywords:** EEG, action perception, social robotics, mirror neuron system, mu rhythm, theta rhythm

## Abstract

The perception of others’ actions supports important skills such as communication, intention understanding, and empathy. Are mechanisms of action processing in the human brain specifically tuned to process biological agents? Humanoid robots can perform recognizable actions, but can look and move differently from humans, and as such, can be used in experiments to address such questions. Here, we recorded EEG as participants viewed actions performed by three agents. In the Human condition, the agent had biological appearance and motion. The other two conditions featured a state-of-the-art robot in two different appearances: Android, which had biological appearance but mechanical motion, and Robot, which had mechanical appearance and motion. We explored whether sensorimotor mu (8–13 Hz) and frontal theta (4–8 Hz) activity exhibited selectivity for biological entities, in particular for whether the visual appearance and/or the motion of the observed agent was biological. Sensorimotor mu suppression has been linked to the motor simulation aspect of action processing (and the human mirror neuron system, MNS), and frontal theta to semantic and memory-related aspects. For all three agents, action observation induced significant attenuation in the power of mu oscillations, with no difference between agents. Thus, mu suppression, considered an index of MNS activity, does not appear to be selective for biological agents. Observation of the Robot resulted in greater frontal theta activity compared to the Android and the Human, whereas the latter two did not differ from each other. Frontal theta thus appears to be sensitive to visual appearance, suggesting agents that are not sufficiently biological in appearance may result in greater memory processing demands for the observer. Studies combining robotics and neuroscience such as this one can allow us to explore neural basis of action processing on the one hand, and inform the design of social robots on the other.

## INTRODUCTION

From dolls and statues, to modern horror and science fiction stories, humans have long been preoccupied with creating other entities in their likeness. Advances in technology now allow us to create increasingly realistic and interactive humanoid agents. Lifelike humanoid robots are becoming commonplace, and assistive technologies based on social robotics are being developed for many application domains (e.g., Kanda et al., 2004; Coradeschi et al., 2006). Research on how humans perceive, respond to and interact with these agents is therefore increasingly important. However little is understood about human social cognition in this new, wider context. An interdisciplinary perspective on social robotics is needed, since this field will impact many areas of research, as well as issues of public concern in the near future, for example in domains such as education and healthcare (Billard et al., 2007; Dautenhahn, 2007; Mataric et al., 2009). Here, we provide hypotheses and data from cognitive and social neuroscience to study the perception of humanoid robots. Our goal is on the one hand to improve our understanding of human social cognition, and on the other, to help engineers and designers develop robots that are well-suited to their application domains.

### ACTION UNDERSTANDING AND THE BRAIN

Understanding the movements and actions of others is critical for survival, and in many species, for social cognition. For humans, these processes are building blocks for important higher-order social skills, such as coordination, communication, intention understanding, and empathy (Blakemore and Decety, 2001; Iacoboni and Dapretto, 2006; Knoblich et al., 2006). A prominent idea regarding how the nervous system achieves the goal of “understanding others” is motor simulation. According to this theory, an action is understood by mapping the visual representation of an observed action to the observers’ own motor representations (Rizzolatti et al., 2001). This view has become more widespread following the discovery of mirror neurons (MNs) in macaque premotor cortex (Di Pellegrino et al., 1992; Gallese et al., 1996; Rizzolatti et al., 1996). MNs are cells that fire both during the execution of an action, and during the observation of the same action performed by another agent, thereby providing a neural basis for motor resonance. For instance a mirror neuron that fires as the monkey cracks a peanut, can also fire as the monkey observes someone else crack a peanut. The neural network in the human brain supporting action and body movement processing is generally referred to as the mirror neuron system (MNS) – sometimes also as action observation network or action perception system – and corresponds to a set of areas in temporal, parietal, and frontal cortices (Rizzolatti et al., 2001; Saygin et al., 2004; Grafton and Hamilton, 2007; Saygin, 2007; Cattaneo et al., 2010; van Kemenade et al., 2012; Cook et al., in press). The MNS received considerable attention in the past two decades as a possible neural basis for action understanding, social cognition, empathy, and communication, and has been discussed in relation to disorders affecting social functions such as autism (Iacoboni and Dapretto, 2006).

Although the majority of studies on human MNS have involved functional magnetic resonance imaging (fMRI) as a method of investigation, there is also a body of evidence from multiple temporally-sensitive methodologies including motor-evoked potentials, magnetoencephalography (MEG), and electroencephalography (EEG) indicating that the motor system is involved during action observation (Fadiga et al., 1995; Hari et al., 1998; Cochin et al., 1999; Babiloni et al., 2002; Pineda, 2005; Hari, 2006; Orgs et al., 2008; Kilner et al., 2009; Perry and Bentin, 2009; Press et al., 2011). EEG studies in particular have revealed another index of human MNS activity known as mu suppression, which can be measured non-invasively via EEG with electrodes placed on the scalp. Mu suppression refers to an attenuation in the power of the EEG in the alpha frequency range (8–13 Hz) measured over sensorimotor cortex and, like mirror neuron activity, is observed both during action execution and action observation (Cochin et al., 1999; Babiloni et al., 2002; Pineda, 2005; Hari, 2006; Orgs et al., 2008; Perry and Bentin, 2009). There is a growing body of literature that is revealing the functional properties of sensorimotor mu suppression. Specifically, it has been suggested that mu suppression might have a role in social interactive contexts in addition to passive action observation (Tognoli et al., 2007; Dumas et al., 2012; Naeem et al., 2012; Silas et al., 2012), and that sub-bands of the mu rhythm might have different functional properties (Naeem et al., 2012). In an attempt to understand the relation between the mu suppression and the MNS, studies using both fMRI and EEG have argued that attenuations in the power of the EEG mu rhythm and fMRI activity in nodes of the MNS likely index the activity of the same underlying neural populations (Arnstein et al., 2011; Braadbaart et al., 2013), although it is worth noting mu suppression has also been correlated with brain areas other than the MNS (Mizuhara and Inui, 2011).

Although the 8–13 Hz oscillations have been the most implicated frequency band in EEG studies of action observation, a thorough understanding of the mechanisms of action observation and of the functional properties of this neural system can benefit from considering other dependent measures whose functional significance in cognition is well studied. As mentioned above, one of the most influential mechanistic explanations of action observation, the motor simulation framework, posits that we understand others’ actions by mapping the visual input of the seen action to our own sensorimotor representations (Rizzolatti et al., 2001). For meaningful actions, during this mapping process, one also needs to activate the existing semantic representations of actions, and compare them with the current visual input and/or the representations evoked during motor simulation (Barresi and Moore, 1996). If there is a match between the seen action’s meaning and existing long-term memory representations, this can result in successful recognition of the action; if there is no match (e.g., in the case of actions or agents that have not been encountered before, and thus do not have a memory trace), the newly encountered item will need to be encoded into long-term memory. Thus, the entire process of action understanding requires the interplay of perceptual, motor, and memory processes.

Although memory is an essential part of action understanding (and the processing of meaningful stimuli in general), most studies to date have approached the issue implicitly (e.g., Umiltà et al., 2001). However, both human behavioral and neuroscience studies (e.g., Stefan et al., 2005; Casile and Giese, 2006; Carmo et al., 2012) and robotics studies (e.g., Wermter and Elshaw, 2003; Ugur and Erol, 2011) have highlighted a role for memory processes in action understanding, and there is growing interest in specifying the role of learning and memory in action perception and related brain systems (Cook et al., in press). EEG theta oscillations have been investigated in the context of memory processes, but have not been studied thoroughly in relation to action understanding. Given the crucial role of memory for action understanding within the motor simulation framework, we believe it is time to incorporate what we know about the functional significance of theta activity in studying action processing. Thus, in the current study, we also explored theta oscillations (4–8 Hz), which, especially at frontal sites, are thought to index memory encoding and retrieval in both linguistic and non-linguistic contexts (Hald et al., 2006; Osipova et al., 2006; Davidson and Indefrey, 2007; Bastiaansen et al., 2008; Shahin et al., 2009; Crespo-Garcia et al., 2010; Klimesch et al., 2010; Zion-Golumbic et al., 2010; Atienza et al., 2011). Specifically, theta activity has been reported to increase during encoding of information into long-term memory, and during retrieval of information from long-term memory (see review Klimesch et al., 2010). Zion-Golumbic et al. (2010) also reported that theta power increase reflects the utilization of information from long-term memory during processing of visual stimuli. Exploration of theta oscillations during action processing could be informative given the automatic employment of memory processing during action observation, and given that there is almost no work on theta oscillations in relation to action observation.

### COGNITIVE NEUROSCIENCE AND ROBOTICS

The cognitive neuroscience of action perception, and especially the MNS, has received intense interest from neuroscientists in the last two decades, and we can now use the accumulated knowledge in this field to study how the human brain supports human-robot interaction. Conversely robotics can help research on the human brain by allowing us to test functional properties of the MNS and other brain areas that support action understanding.

One question that has been of interest since the identification of the MNS is whether the system is selectively tuned to process the actions of biological agents. For example, we may ask, during perception of or interactions with robots, does the brain rely on the same or distinct processes as with perception of or interactions with biological agents? The neuroscience-based theory of motor simulation argues that a visually perceived body movement or action is mapped onto the perceiving agent’s sensorimotor neural representations, and “an action is understood when its observation causes the motor system of the observer to ‘resonate’” (Rizzolatti et al., 2001). But what are the boundary conditions for “resonance?” What kinds of agents or actions lead to the simulation process? Is biological appearance important? Is biological motion? Artificial agents such as robots can be important experimental stimuli to test such hypotheses since robots can perform recognizable actions like biological agents, but can differ from biological agents in some other aspects (e.g., on how they appear or how they move – see below).

The neuroscience literature on the perception of robots has not revealed consistent results (Kilner et al., 2003; Chaminade and Hodgins, 2006; Chaminade et al., 2007; Gazzola et al., 2007; Oberman et al., 2007; Press et al., 2007). Some studies have reported that artificial agents’ actions apparently affect the observers’ own motor processing, or activity within the MNS, whereas others have argued that the MNS either does not respond, or responds weakly if the perceived actor is not human, including a clear claim that the MNS is only “mirror” for biological actions (Tai et al., 2004).

Conversely, neuroscience research on human observation of and interaction with robots can be invaluable to social robotics researchers since an important issue in the growing field of personal and social robotics is how to design robots that are likely to be socially accepted by their human companions. Research on the neural basis of social cognition using robots can provide valuable insights to advance the field of robot design and human-robot interaction by identifying the critical qualities that a robot should have, and eventually to guide the building of “neuroergonomic” robots that people are comfortable to interact with (Saygin et al., 2011).

### BRAIN ACTIVITY AND ROBOT DESIGN

Here, we explored human brain activity evoked by humans and robots. Robots can have a range of appearance and movement patterns – but at the same time, they can be perceived as carrying out recognizable actions. Is biological appearance or biological movement necessary for engaging human brain systems that support social cognition? Does robot perception require additional memory processing demands? Robots can allow us to ask such questions and to test whether particular brain systems are selective for or sensitive to the presence of a human, or an agent with a humanlike form, or whether they respond similarly regardless of the agent performing the action.

Given that action observation is important for imitation learning and higher-level social skills, we hypothesized that human likeness of the observed agent (i.e., the degree of similarity between the observer and the observed agent) could be important for the MNS. Indeed, motor resonance theory would predict increased humanlikeness would lead to more effective or efficient simulation (e.g., Buccino et al., 2004; Calvo-Merino et al., 2006; Casile et al., 2010). On the other hand, in artificial agents, human resemblance is not necessarily always a positive feature. The “uncanny valley” (UV) hypothesis suggests that as a robot is made more humanlike, the reaction to it becomes more and more positive, until a point is reached at which the robot becomes oddly repulsive (Mori, 1970). This phenomenon is well known to roboticists and animators, but its scientific understanding remains incomplete – although there is a growing body of research on the topic, with some recent contributions from the behavioral and neural sciences (e.g., MacDorman and Ishiguro, 2006; Ho et al., 2008; Steckenfinger and Ghazanfar, 2009; Cheetham et al., 2011; Thompson et al., 2011; Tinwell et al., 2011; Lewkowicz and Ghazanfar, 2012; Saygin et al., 2012).

Most studies on the observation of robot actions have used very basic robot arms consisting of a stick/body and a claw, akin to rudimentary industrial robot arms, performing grasping, or other simple movements. Therefore, the results are not sufficient to make conclusions regarding social humanoid robots that are being developed today. To overcome these limitations of previous work, we created well-controlled stimuli based on state-of-the-art humanoid robots developed by an interdisciplinary team. Furthermore, our hypotheses, stimuli, and experimental design focused on whether the seen agent had biological (humanlike) appearance, whether the agent’s body movements were biological, plus whether their appearance and movements matched (Saygin et al., 2012).

**FIGURE 1:**
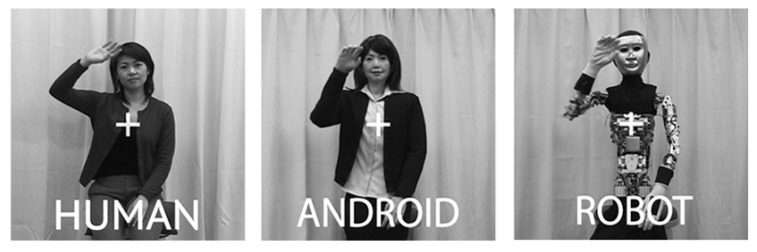
**Still frames from the videos used in the experiment depicting the three actors: Human, Android, and Robot**.

We used human EEG cortical oscillatory activity in the alpha/mu and theta frequency bands as dependent measures in the present study. In addition to asking functional questions about action processing and social cognition, we also hoped to shed new light onto the functional significance of these dependent measures in relation to action observation. For instance, are cortical theta and mu oscillations sensitive to the sensory properties of the stimuli, or to higher-level cognitive processes? In particular, we investigated whether cortical theta and mu oscillations are modulated by the human likeness of the observed agent. We characterized human likeness in two different ways: in terms of appearance and in terms of motion. Participants watched videos of three agents as their EEG was recorded: Human, Android, and Robot. Human had biological appearance and movement, Android had biological appearance and mechanical movement, and Robot had mechanical appearance and mechanical movement (see **Figure 1**, Methods, and Saygin et al., 2012 for more detail).

We hypothesized that if mu suppression is influenced by the specific visual properties of the seen action, we might find a difference between the actions of the different agents based on their appearance and/or motion characteristics. If on the other hand mu suppression reflects higher-level processes related to the meaning of the action, then the agents might not differ from each other since they all perform the same recognizable actions despite their different degrees of human likeness. For theta activity, we hypothesized that its power would be modulated by the human likeness of the observed agent, reflecting the processing demands of mapping the visual input into existing semantic representations. Since in the context of action processing, people are more familiar with human actors than robot actors, we hypothesized memory processes would differ depending on the agent’s appearance. More specifically, we hypothesized that the power of the theta oscillations would decrease as a function of the human likeness of the observed agent, since observation of relatively unfamiliar stimuli would result in greater memory processing demands (Hald et al., 2006; Zion-Golumbic et al., 2010; Atienza et al., 2011). We thus expected that observation of the Robot would result in increased theta activity compared to the Human, since the humanlike appearance of the agent would facilitate access to semantic representations related to human action. The Android condition, which features humanlike appearance but non-human motion, additionally allows us to ask whether or not the human likeness of the motion is a modulator of memory processes.

In sum, the aim of the study was threefold. First, by manipulating various features of the observed agent, we aimed to improve our understanding of the functional significance of EEG mu and theta oscillations during action observation and their relation to the MNS. Second, using robots as experimental stimuli in the presence of existing knowledge in cognitive neuroscience of action perception, we aimed to inform robotics about how humans respond to robots of varying degrees of human likeness, what dependent measures could be used as gold-standards for social robotics research, and accordingly for guiding the design of robots in the long-term. Finally, the current study allowed us to do cross-methodology comparison, as we previously reported an fMRI study utilizing the same agents as stimuli (Saygin et al., 2012).

## MATERIALS AND METHODS

### PARTICIPANTS

Twelve right-handed adults (three females; mean age = 23.4; SD = 4.7) from the student community at the University of California, San Diego participated in the study. Participants had normal or corrected-to-normal vision and no history of neurological disorders. We recruited only those participants who had no experience working with robots in order to minimize possible effects of familiarity or expertise on our results (MacDorman et al., 2009). Informed consent was obtained in accordance with the UCSD Human Research Protections Program. Participants were paid $8 per hour or received course credit.

### STIMULI

Stimuli were video clips of actions performed by the humanoid robot Repliee Q2 (in robotic and humanlike appearance, **Figure 1** right and middle images, respectively) and by the human “master,” after whom Repliee Q2 was modeled (**Figure 1** left image). We refer to these agents as the Robot, the Android (dressed up robot), and the Human conditions (even though the former two are in fact the same robot).

Repliee Q2 has 42 degrees of freedom and can make face, head, and upper body movements (Ishiguro, 2006). The robot’s movements are mechanical or “robotic,” and do not match the dynamics of biological motion. The same movements were videotaped in two appearance conditions. For the Robot condition, Repliee Q2’s surface elements were removed to reveal its wiring, metal arms, and joints, etc. The silicone “skin” on the hands and face and some of the fine hair around the face could not be removed but was covered. The movement kinematics for the Android and Robot conditions was identical, since these conditions comprised the same robot, carrying out the very same movements. For the Human condition, the female adult whose face was used in constructing Repliee Q2 was videotaped performing the same actions. All agents were videotaped in the same room with the same background. Video recordings were digitized, converted to grayscale and cropped to 400 × 400 pixels. Videos were clipped such that the motion of the agent began at the first frame of each 2 s video.

In summary, we had three agents and varied the form and motion of the observed agent: a human with biological appearance and motion, an Android with biological appearance and mechanical motion, and a Robot with mechanical appearance and motion. Due to the considerable technical difficulty in developing these stimuli and limitations inherent to the robot systems we worked with, we did not have a fourth condition (i.e., an agent with a well-matched mechanical appearance and biological motion) that would make our experimental design 2 (motion) × 2 (appearance).

### PROCEDURE

Before starting EEG recordings, participants were presented with all the action stimuli and were informed as to whether each agent was human or robot. Since prior knowledge can induce cognitive biases against artificial agents (Saygin and Cicekli, 2002), each participant was given exactly the same introduction to the study. Participants went through a short practice session before the experiment.

EEG was recorded as participants watched video clips of the three agents performing five different upper body actions (drinking from a cup, picking up and looking at an object, hand waving, introducing self, nudging). The experiment consisted of 15 blocks of 60 trials with equal number of videos of each agent and action (four repetitions of each video in each block). Stimuli were presented in a pseudo-randomized order ensuring that a video was not repeated on two consecutive trials. Each participant experienced a different pseudo-randomized sequence of trials.

Stimuli were displayed on a 22″ Samsung LCD monitor at 60 Hz using Python-based Vizard (Worldviz, Inc.) software. We displayed a gray screen with a fixation cross before the start of the video clip on each trial. Participants were instructed to fixate the blue fixation cross at the center of the screen for 700–1000 ms. Then the color of the fixation cross was changed to green and presented for 500–700 ms to inform participants of the upcoming video. A comprehension question was displayed every 6–10 trials after the video, asking participants a true/false question about the action in the just seen video (e.g., Drinking?). Since participants did not know whether they would receive a question during video presentation, this task allowed us to direct the subjects’ attention to the stimuli, but not in a manner that might bias the results for any particular condition (behavioral performance in the task did not differ across conditions; all *p* values > 0.1). Participants responded with a bimanual key press (Yes/No responses).

### EEG RECORDING AND DATA ANALYSIS

EEG was recorded at 512 Hz from 64 Active Two Ag/AgCl electrodes (Biosemi, Inc.) following the International 10/20 system. The electrode-offset level was kept below 25 k ohm. Four additional electrodes were placed above and below the right eye, and lateral to the eyes to monitor oculomotor activity. Two mastoid electrodes were placed behind the ears for re-referencing. The data were preprocessed with MATLAB and the EEGLAB toolbox (Delorme and Makeig, 2004). Each participant’s data were first high-pass filtered at 1 Hz, low-pass filtered at 50 Hz, and re-referenced to average mastoids. Then the data were epoched ranging from 900 ms preceding video onset to 2000 ms after video onset, and were time-locked to the onset of the video clips. Atypical epochs of electromyographic activity were removed from further analysis by semi-automated epoch rejection procedures (kurtosis and probability-based procedures with standard deviation ≥ 6). To remove eye-related artifacts, the data were decomposed by extended infomax ICA using the algorithm *binica*, and components that showed typical eye-related artifact characteristics were removed from the data. After preprocessing, data for each condition were transformed into a spectrographic image using 3-cycle Morlet wavelets in the 4–55 Hz frequency range at a number of frontal channels (F3 and F4), central channels (C3 and C4 over the sensorimotor cortex), and parietal channels (P3 and P4). The frontal and central channels were selected since these or neighboring electrodes were consistently reported in the literature on theta and mu oscillations, respectively (Hald et al., 2006; Oberman et al., 2007; Zion-Golumbic et al., 2010). For both mu and theta oscillations, these are the specific regions of interest that are related to our hypotheses regarding MNS and memory, and posterior electrodes for each frequency band are believed to have different functional significance. However, for completeness, we reported also on parietal channels to cover the posterior parts of the scalp. The mean power of the baseline period of the spectrographic images was removed from the power at each time point of the experimental trials.

### STATISTICAL ANALYSIS

The spectral windows of mu and theta oscillations for statistical analyses were determined from the mean spectrographic images across all conditions in the 4–55 Hz frequency range and constrained by well-established windows of these cortical rhythms, which are 8–13 Hz for mu and 4–8 Hz for theta. The specific time windows for statistical analyses of the power of mu and theta oscillations were determined from the mean spectrographic image across all conditions, allowing us to test modulations in time periods of interest without introducing any bias for finding specific condition differences. For mu, mean alpha power in the time window of the mu attenuation (400–1400 ms after stimulus onset) was extracted for each condition (Agent) and channel (C3: left hemisphere; C4: right hemisphere), and entered into a 3(Agent) × 2 (Hemisphere) repeated measures ANOVA. For theta, the mean power in the time window of the theta increase (150–400 ms after stimulus onset) was extracted for each condition (Agent) and channel (F3: left hemisphere; F4: right hemisphere) and entered into a 3(Agent) × 2 (Hemisphere) repeated measures ANOVA. Although our hypotheses primarily related to the Agent manipulation (Robot, Android, Human), we also modeled Action (the five different actions) and Hemisphere (left, right) to explore any modulation that may be specific to particular actions. These analyses are not reported since they did not reveal any action-specific effects or interactions, and the effects reported below for the 3 × 2 ANOVA did not change. Greenhouse–Geisser correction was applied to the ANOVAs whenever indicated. *p*-values reported below are two-tailed except for the comparisons of mu and alpha power against zero, where our hypotheses were one-tailed (i.e., we expected a decrease in mu power and an increase in theta power). Planned or posthoc *t*-test *p*-values were corrected for multiple comparisons.

In addition to our hypothesis-driven ANOVAs described above, for completeness, we also included ANOVAs for each of theta and mu oscillations in the other channel locations: (C3, C4) and (P3, P4) for theta; (F3, F4) and (P3, P4) for mu. Furthermore, given recent experimental evidence that sub-bands of the mu band might have different functional properties (Naeem et al., 2012), we ran two additional 3(Agent) × 2 (Hemisphere) ANOVAs for lower (8–10 Hz) and upper (10–13 Hz) bands of the mu oscillations at channels C3 and C4.

### MULTIVARIATE PATTERN ANALYSES

In recent years, computational methods from machine learning have been used to analyze neuroimaging data as an alternative to conventional analyses (Kamitani and Tong, 2005; Haynes and Rees, 2006; Norman et al., 2006). The idea is to build a model (classifier) that can decode information recorded from the brain with neuroimaging. This is done by first training the model with a set of data labeled with class information (e.g., the conditions of the experiment) and allowing it to learn the patterns within the data, and then testing it with a separate set of data to see whether it can correctly predict unlabeled data. Predictions with higher-than-chance accuracy indicate that there is sufficient information in the data that distinguishes the neural patterns corresponding to different conditions of an experiment. The advantage of these methods is that they are more sensitive to the differences between conditions since they consider the patterns of activity as the basic units of measurement, as opposed to an average of the activity, which may discard useful information. This is important in the context of the current study since there are discrepancies in the mu suppression literature, which might be due to the information lost by using the traditional analysis (i.e., averaging technique).

In order to explore subtle differences that may be missed when analyzing mu and theta oscillations with traditional analyses as described above, we used Multivariate Pattern Analysis (MVPA) using the pattern of mu activity and pattern of theta activity. We used support vector machines (Cortes and Vapnik, 1995) with a linear basis function and the LIBSVM software package (Chang and Lin, 2011) on mu oscillations at channels C3 and C4, and theta oscillations at channels F3 and F4 in three-way [Robot-Android-Human (R-A-H)] and two-way classifications [Robot-Android (R-A), Robot-Human (R-H), Android-Human (A-H)]. The data that were fed into the classifier were time-frequency features in the frequency range 8–13 Hz and in the time interval 400–1400 ms for mu, and time-frequency features in the frequency range 4–8 Hz and in the time interval 150–400 ms for theta. The data were scaled before classification and five-fold cross validation was applied in the classification procedure. The prediction accuracy (the number of correctly predicted trials) was used as the performance metric of the classifier. Each classification (R-A-H, R-A, R-H, A-H) was run three times for each subject and the average prediction accuracy of these three runs are reported. Above-chance performance (corresponding to the 95% confidence interval) was 54.37% for the two-way classifications, and 37.59% for the three-way classification (Muller-Putz et al., 2008).

## RESULTS

### MU OSCILLATIONS (8–13 Hz)

**FIGURE 2:**
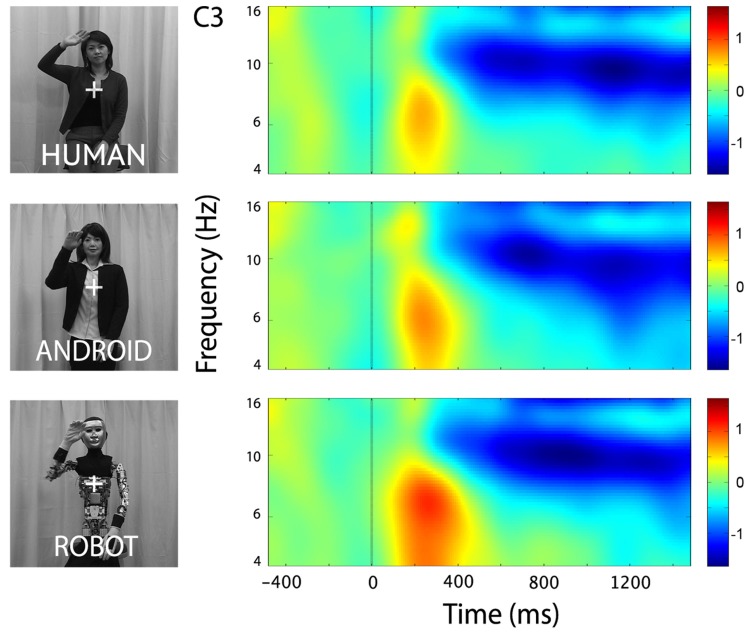
**Time-frequency plots for the three conditions (Human, Android, Robot) at channel C3 (left hemisphere).** Plots for the right hemisphere (C4) were very similar and are not shown. The frequency axis is log scaled. The zero point on the time axis indicates the onset of the action movies. Shortly after the onset of the action videos, we observed an increase in the theta frequency band (see also **Figure 4**), followed by an attenuation in the alpha frequency band (8–13 Hz) that started around 350 ms, and grew stronger around 600 ms.

**FIGURE 3:**
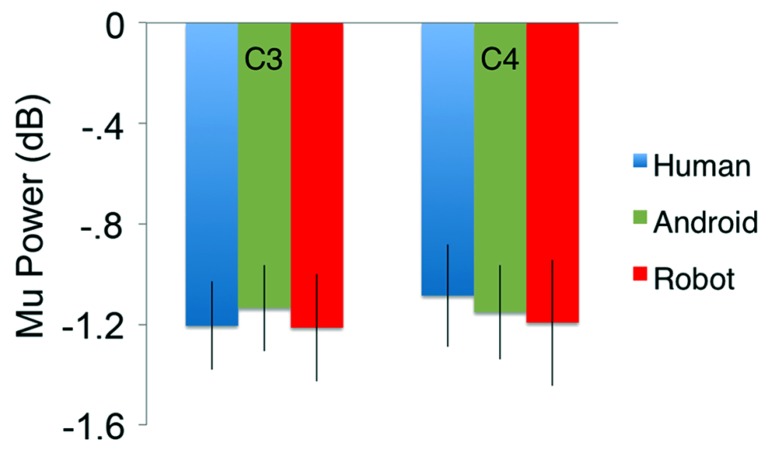
**Attenuation in the power (in dB) of the mu (8–13 Hz) oscillations for the three conditions (Human, Android, Robot) plotted at channels C3 and C4.** Error bars indicate the standard error of the mean. For both C3 and C4, all conditions led to statistically significant attenuation in mu power (all *p*’s < 0.05, see Results). There were no significant differences between agents (Human, Android, Robot) or hemispheres (C3, C4).

In the channels of interest, C3 and C4, action observation led to an increase in theta power shortly after stimulus onset (see theta results below for quantified analyses), followed by an attenuation in alpha power starting around 350 ms, and becoming stronger around 600 ms after stimulus onset (**Figure 2**). For observation of all agents’ actions (Human as well as the two robot agents, Android and Robot), attenuation of the mu oscillations were robust and significant (**Figure 3**; C3: Human (Mean = -1.21, SD = 0.61), *t*(11) = -6.871, *p* < 0.001; Android (Mean = -1.14, SD = 0.60), *t*(11) = -6.642, *p* < 0.001; Robot (Mean = -1.21, SD = 0.74), *t*(11) = -5.675, *p* < 0.001, and C4: Human (Mean = -1.09, SD = 0.71), *t*(11) = -5.328, *p* < 0.001; Android (Mean = -1.15, SD = 0.65), *t*(11) = -6.11, *p* < 0.001; Robot (Mean = -1.19, SD = 0.87), *t*(11) = -4.76, *p* = 0.001). Suppression in alpha power was also observed in frontal and parietal channels over the scalp with greater suppression at parietal channels. Although, we report some results from other channels here for descriptive purposes, given the differential functional significance of frontal and posterior alpha, our focus will be on the hypothesis-driven analyses at channels C3 and C4.

#### ANOVA

Our primary comparison of interest was the 3(Agent) × 2 (Hemisphere) repeated measures ANOVA at central channels C3 and C4, which revealed no main effect of Agent [*F*(2, 22) = 0.151] or Hemisphere [*F*(1, 11) = 0.163] on the power of the mu oscillations (all *p* > 0.1; **Figure 3**). There was no Agent × Hemisphere interaction [*F*(2, 22) = 0.947, *p* > 0.1].

When we explored lower (8–10 Hz) and upper (10–13 Hz) bands of the mu oscillations at the same channels separately, we again found no main effects or interactions {Lower Mu: Agent [*F*(1.376, 15.136) = 0.047], Hemisphere [*F*(1, 11) = 0.007], Agent × Hemisphere [*F*(2, 22) = 1.093]; Upper Mu: Agent [*F*(2, 22) = 0.216], Hemisphere [*F*(1, 11) = 0.136], Agent × Hemisphere [*F*(2, 22) = 0.496] all *p* > 0.1}.

Similar 3(Agent) × 2 (Hemisphere) repeated measures ANOVAs at frontal (F3, F4) and parietal channels (P3, P4) are reported here for completeness: There were no main effects or interactions {F3-F4: Agent [*F*(2, 22) = 0.210], Hemisphere [*F*(1, 11) = 0.110], Agent × Hemisphere [*F*(2, 22) = 1.334]; P3-P4: Agent [*F*(2, 22) = 0.629], Hemisphere [*F*(1, 11) = 1.187], Agent × Hemisphere [*F*(2, 22) = 0.359], all *p* > 0.1}.

#### Multivariate pattern analysis

Multivariate pattern analyses of the mu suppression at channels C3 and C4 were performed to reveal any subtle modulations in alpha power over time that may have been missed due to averaging in the traditional analysis. For the three-way classification R-A-H, the average performance of MVPA for all subjects was not above chance (33.91% for C3 and 34.28% for C4). Pairwise classifications R-A, R-H, and A-H also resulted in chance-level performance on average (50.53, 52.11, and 49.77%, respectively for channel C3, and 50.95, 51.31, and 50.82%, respectively for channel C4).****

### THETA OSCILLATIONS (4–8 Hz)

**FIGURE 4:**
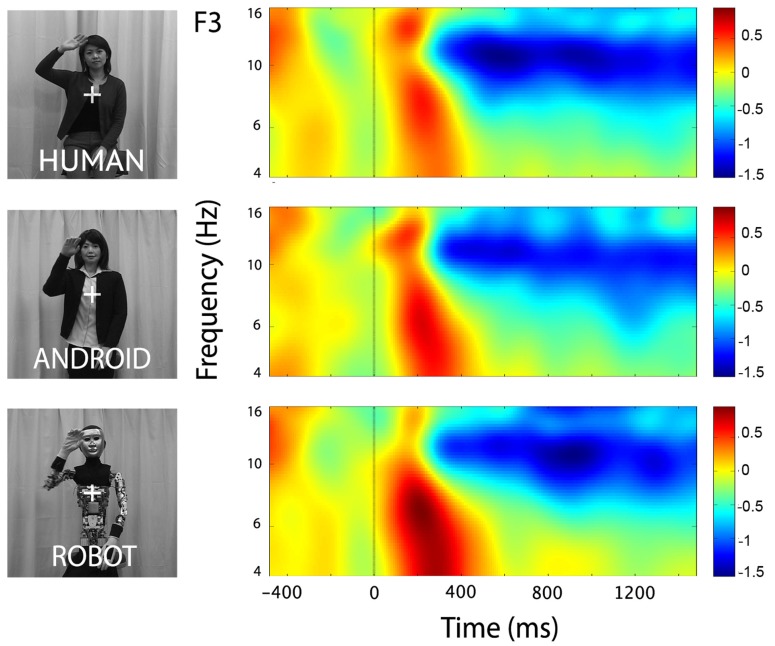
**Time-frequency plots for the three conditions (Human, Android, Robot) at channel F3 (left hemisphere).** Plots for the right hemisphere (F4) were very similar and are not shown. The frequency axis is log scaled. The zero point on the time axis indicates the onset of the action movies. Shortly after the onset of the stimuli, there was an increase in theta power (4–8 Hz), followed by a reduction in alpha power (see also **Figure 2**).

At channels F3 and F4, action observation led to an increase in theta power starting at around 150 ms and lasting until about 400 ms after stimulus onset (**Figure 4**), followed by an attenuation in alpha power (see Mu results above for quantified analyses). For the Robot condition, the increase in theta was significant at both F3 and F4 [**Figure 4**; For F3, Mean = 0.71, SD = 1.05, *t*(11) = 2.322, *p* < 0.01; for F4, Mean = 0.83, SD = 1.13, *t*(11) = 2.527, *p* < 0.01]. Observation of Android and Human actions also resulted in increased theta power that were either statistically significant or just at the cusp of significance (**Figure 4**; For F3, Human (Mean = 0.32, SD = 0.75), *t*(11) = 1.479, *p* = 0.054; Android (Mean = 0.45, SD = 0.88), *t*(11) = 1.774, *p* = 0.05; For F4, Human (Mean = 0.37, SD = 0.68), *t*(11) = 1.848, *p* < 0.05; Android (Mean = 0.37, SD = 0.84), *t*(11) = 1.506, *p* = 0.053). Increase in the power of theta oscillations was also observed at central and parietal channels over the scalp. Although, we reported results from all channels here, we based our discussion mainly on the hypothesis-driven results at channels F3 and F4 given the prior literature.

#### ANOVA

**FIGURE 5:**
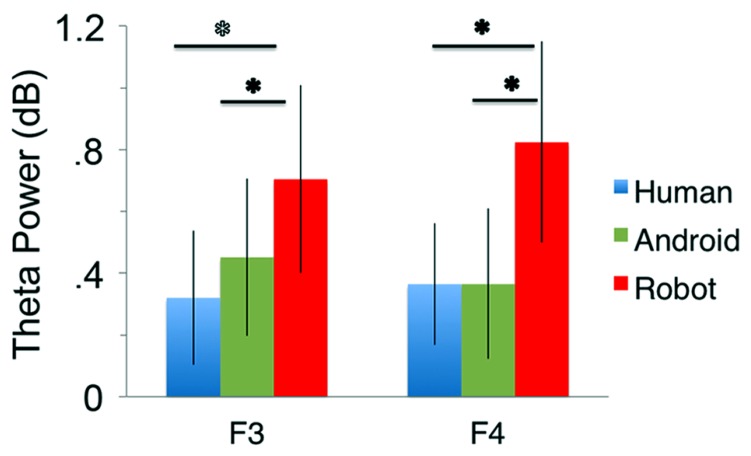
**Power in the theta frequency range (4–8 Hz, in dB) for the three conditions (Human, Android, Robot) plotted at channels F3 and F4.** Error bars indicate the standard error of the mean. All conditions led to significant increase in theta power (all *p*’s í 0.05, see Results). The Robot condition led to significantly increased theta power in comparison to the Android and Human conditions (*p’s í 0.05, see Results).

Our main comparison of interest, a 3(Agent) × 2 (Hemisphere) repeated measures ANOVA at channels F3 and F4 revealed a significant main effect of Agent [*F*(1.350, 14.852) = 5.276, *p* < 0.05, see **Figure 5**]. Planned comparisons (paired *t*-tests) indicated theta oscillations were greater for the Robot condition compared with the Human [*F*(1, 11) = 5.386, *p* < 0.05] and the Android conditions [*F*(1, 11) = 9.879, *p* < 0.01]. The effect of Hemisphere [*F*(1, 11) = 1.144, *p* > 0.1] or the Agent × Hemisphere interaction [*F*(1, 11) = 3.196, *p* > 0.1] were not significant.

Similar 3(Agent) × 2 (Hemisphere) repeated measures ANOVAs at central and parietal channels are reported here for completeness: There was a main effect of Agent at central channels, but no effect of Hemisphere or interaction effect {C3-C4: Agent [*F*(1.133, 12.458) = 5.016], *p* < 0.04, Hemisphere [*F*(1, 11) = 0.401], *p* > 0.1, Agent × Hemisphere [*F*(2, 22) = 1.819]}. The Agent effect reflected increased theta for the Robot, similar to that found in frontal channels (see **Figure 4**). There were no main effects or interactions in parietal channels {P3-P4: Agent [*F*(1.260, 13.860) = 2.588], Hemisphere [*F*(1, 11) = 1.078], Agent × Hemisphere [*F*(2, 22) = 0.908], all *p* > 0.1}.

#### Multivariate pattern analysis

Although traditional analyses already revealed differences between agents, we applied multivariate pattern analyses on the theta oscillations at channels F3 and F4 for completeness. For the three-way classification R-A-H, the average performance of MVPA for all subjects was above chance (39.58% for C3 and 39.53% for C4). Pairwise classifications R-A and R-H resulted in above-chance performance on average (58.25 and 58.33%, respectively for channel F3, and 57.80 and 58.61%, respectively for channel F4). A-H classification resulted in chance-level performance on average (51.76% for channel F3 and 52.16% for channel F4). These MVPA results were thus in line with the results of the traditional analyses.

## DISCUSSION

We investigated how the sensorimotor EEG mu rhythm that is considered to index human MNS activity, and the frontal theta activity that is implicated in memory processes are modulated by the human likeness of the agent being observed. Participants viewed three agents, a Human, and a state-of-the-art robot in two different appearances (as an Android and a Robot) performing the same recognizable actions. The Human had biological motion and appearance, whereas the Android had biological appearance and mechanical motion, and the Robot had mechanical motion and mechanical appearance (**Figure 1**). We hypothesized that any modulations of the oscillations by sensory features of the stimuli would be revealed as significant differences between the experimental conditions, based on the seen agents’ differing appearance and motion characteristics. Specifically if these dependent measures are sensitive to the movement kinematics of the seen actor, then we would expect the Human condition to be distinguished from the others. If they are sensitive to the appearance, then the Robot would be distinguished from the other agents, or there would be a degree of activity that corresponds to the degree of human likeness of the appearance of the agents. If they are sensitive to the congruence of the movement dynamics and appearance, then Android would be distinguished from the other agents since this condition features a humanlike appearance along with non-human motion dynamics, whereas the other agents feature congruent appearance and motion (both biological, or both mechanical). If on the other hand these dependent measures reflect higher-level processing related to the meaning of the actions and are not sensitive to the visual properties of the stimuli, then the agents might not differ from each other since they all perform the very same actions.

### Mu OSCILLATIONS

We showed that the observation of the human agent as well as both of the robot agents resulted in robust and significant attenuations in the power of mu oscillations over the frequently reported sensorimotor areas. The magnitude of the attenuations was equivalent for all agents. This replicates and extends a previous mu suppression study that had used a simple robot hand (Oberman et al., 2007). Consistent with previous work on action observation, we did not find any hemispheric differences (Babiloni et al., 2002). Overall, our results show that the human MNS is unlikely to be selective only for other humans, since a commonly accepted measure of human MNS activity (EEG mu suppression) showed robust and significant modulations also when observing robot actions. These data also suggest that mu suppression might not be sensitive to early sensory stages of action processing, since the agents’ differences in terms of their visual appearance and movement kinematics did not differentially affect mu power. Frontal and parietal sites also showed the same pattern of results as the sensorimotor channels, although it must be noted that alpha oscillations at these latter sites are not specifically linked to the MNS or action processing.

After exploring mu suppression with traditional statistical analyses adopted from previous work (e.g., Oberman et al., 2007), we also explored the data using machine learning and multivariate pattern analyses. The pattern activity has more information than the average activity (over time and frequency band) used in traditional analyses so more subtle differences can be picked up (see Kamitani and Tong, 2005; Norman et al., 2006; Pereira et al., 2009 for discussion of such issues). Our primary goal in applying pattern analysis on mu oscillations was to reduce concerns readers may possibly have about lack of a difference between conditions being due to an insensitive analysis method. In other words, we wanted to pre-answer the question a reader may have about whether there could be subtler differences when the entire pattern gets taken into account, especially given that recent literature on the mu suppression field has started to include finer modulations (Naeem et al., 2012). The fact that we did not find differences in the patterns of mu suppression with this much more sensitive analysis method provides strong evidence that mu suppression is also found for observing the actions of humanoid robots. Mu suppression patterns do not appear to be sensitive to the early sensory stages of action processing (as evidenced by chance-level performance for the R-A-H classification), in particular to the appearance (as evidenced by chance-level performance for the R-A classification) or the movement kinematics (as evidenced by chance-level performance for the A-H classification) of the observed agent.

To be clear, there may be other systems in the brain that are modulated by sensory properties of the seen stimuli, or even those that are selective for processing biological agents. Indeed, in related work (and in the theta results here), we have reported perceptual and neural processes that are sensitive to the properties of the seen action such as humanlike appearance or motion (e.g., Saygin and Stadler, 2012; Urgen et al., 2012). The mu suppression results here indicate however that the human MNS does not appear to respond differentially to the actions of humanoid robots and humans. Although a PET study had claimed the human MNS is “mirror only for biological actions” (Tai et al., 2004), several recent fMRI studies are consistent instead with our present results, and have reported that human MNS also responds to robot actions (e.g., Gazzola et al., 2007; Cross et al., 2012; Saygin et al., 2012). In particular, Saygin et al. (2012), using very similar stimuli to the present study, found no difference between human and robot actions in premotor cortex, but showed that parietal cortex is sensitive to the congruence of the motion and appearance of the agent (as evidenced by significant differences in response to the Android). More broadly, these data are consistent with the view that the premotor cortex is largely insensitive to the surface properties of the stimuli depicting actions, but instead is more involved in computing goals and intentions (Rizzolatti et al., 2001; Grafton and Hamilton, 2007; Cattaneo et al., 2010). Human fMRI studies indicate that human premotor cortex responds to a wide range of action stimuli, including impoverished or simplified displays such as point-light biological motion or simple avatars (Pelphrey et al., 2003; Saygin et al., 2004). Since the mu rhythm appears to be insensitive to the visual aspects of the actions (i.e., the humanlike appearance and movement kinematics in the current study), cognitive and affective manipulations during passive action observation or social interactive contexts as evidenced by recent literature (Tognoli et al., 2007; Dumas et al., 2012; Naeem et al., 2012; Silas et al., 2012) would be more appropriate for future studies to better understand the functional properties of the mu rhythm. The fact that we did not find any difference between the different sub-bands of the mu rhythm further suggests that social interactive contexts may be suitable to study the functional properties of the mu rhythm (Naeem et al., 2012).

### THETA OSCILLATIONS

For the frontal theta oscillations, we expected our meaningful stimuli to lead to increases in power, reflecting memory-related processing (i.e., accessing long-term memory representations to process the higher-level meaning of the action stimuli). In particular, we hypothesized that the power would decrease as a function of the human likeness of the observed agent, since observation of relatively unfamiliar agents could result in greater memory processing demands (Hald et al., 2006; Zion-Golumbic et al., 2010; Atienza et al., 2011). More specifically, we hypothesized that observation of the Robot would result in greater theta activity compared to the Human, as we expected the humanlike appearance of the agent would facilitate access to semantic representations related to human action. However, it was also possible biological motion would also influence responses, in which case we would expect the Android condition to also differ from the Human.

Our analysis of the frontal theta activity indeed showed that observation of the Robot resulted in a significantly stronger increase in the power of theta oscillations (4–8 Hz) compared to the agents with humanlike appearance; the Human and Android did not differ from each other. MVPA of the theta oscillations corroborated these results. Since the Robot was distinguished from the other agents by its non-human appearance, these results suggest that frontal theta activity is modulated by the appearance of the agent being observed during action observation. Central sites revealed a similar pattern of results with the frontal sites; there were no agent differences over parietal sites.

Since theta oscillations reflect memory processes such as retrieval from long-term memory and encoding into long-term memory (see review Kahana et al., 2001; Klimesch et al., 2010), our results suggest that processing of the Robot resulted in greater demands on memory systems as compared to the other agents. This difference is best viewed as reflecting the interplay of perceptual processing and long-term memory, particularly during retrieval of items from semantic memory. A robotic appearance, especially in the context of actions that are typical for humans, is less likely to be associated with strong semantic links that can aid in the mapping of the visual input onto existing representations from long-term memory. The difficulty of integrating the visual input with existing semantic knowledge could manifest itself as increased frontal theta activity in comparison to the conditions with humanlike appearance. For the human stimuli, linking the visual input with semantic representations of human actions is likely to be less effortful, since participants have had existing semantic representations about actions developed over time by seeing other humans. This interpretation is consistent with previous work, which has found increased theta activity during the retrieval of semantic information from long-term memory, and especially sensitivity to semantic congruence in linguistic and non-linguistic contexts (Hald et al., 2006; Davidson and Indefrey, 2007; Bastiaansen et al., 2008; Shahin et al., 2009; Zion-Golumbic et al., 2010; Atienza et al., 2011; Steele et al., 2013). The similarity of the results for the Android with that of the Human suggests that the very humanlike appearance of the Android may have facilitated the activation of semantic representations about human actions, even though the motion of this agent was not humanlike (and was in fact the same as that for Robot), and even though participants knew that this agent was not a real human.

In a recent event-related potential (ERP) study (Urgen et al., 2012), we averaged the EEG time-locked to the onset of actions for the Human, Android, and Robot conditions. While all action stimuli evoked a significant negativity called the N300/N400 component complex beginning at around 200 ms after stimulus onset over frontal channels, the amplitude of this component differed significantly for the Robot condition compared to the other agents, a parallel to the present results. Given the timing of the theta oscillations observed here, and the known function of these ERP components for semantic processing (Sitnikova et al., 2008; Kutas and Federmeier, 2011; Wu and Coulson, 2011), we conclude that a humanlike appearance facilitates (or a non-human appearance impedes) access to long-term memory representations related to action. The link between frontal theta and ERP components related to memory processes should be explored in future work. Furthermore, the addition of a condition that presents a biological motion and mechanical appearance combination can be useful to better understand the interaction between the appearance and motion parameters.

### CROSS-METHODS COMPARISON: EEG AND fMRI WITH HUMAN AND ROBOT ACTION STIMULI

The present study allows us to compare our EEG time-frequency results to our previous fMRI work with a similar stimulus set (Saygin et al., 2012). The main finding of our fMRI study was that parietal regions of the human cortex (specifically bilateral anterior intraparietal sulcus, which is part of the human MNS) responded significantly more to the Android agent, therefore to the mismatch of form and motion of the agent being observed. Premotor regions of the MNS did not show selectivity for the form or the motion of the agents. Although EEG mu activity has been found to correlate with fMRI activity both in premotor cortex and parietal cortex (Arnstein et al., 2011), our studies suggest that the mu rhythm might share more functional properties with the activity of premotor cortex than parietal cortex.

In the current study theta oscillations distinguished the Robot agent around 150–400 ms after stimulus onset. Although there was a region in left lateral temporal cortex (the extrastriate body area) that responded significantly less to the Robot agent in the fMRI data, based on the functional properties of this region, this activation is more likely to reflect visual stimulus properties rather than the memory-related processing indexed by the theta oscillations in the present study, or by event related potentials (Urgen et al., 2012). It is likely that EEG, with its milliseconds time resolution, can reveal effects that do not emerge in fMRI studies due to the limited time resolution of this latter method.

### HUMAN QUALITIES OF SOCIAL ROBOTS

Neuroscience research on human observation of and interaction with robots not only improves our understanding of the neural basis of social cognition but it can be invaluable to social robotics researchers. One important issue in the growing field of personal and social robotics is how to design robots that are likely to be socially accepted by their human companions. Broadly, there are two important design issues. The first is the visual properties of robots, i.e., how they should look on the surface. The second is the functional properties, i.e., how they should perform the functions they are designed to perform. Not only should we consider both visual properties and functional properties, but the combination (or interaction) of them might also be important for determining the eventual social acceptability of a robot. Therefore, research efforts for robotics from social, behavioral, and neural sciences should focus on both of these aspects. In the present study, we kept the functioning of the robots constant (i.e., both robots successfully performed various goal-directed, recognizable human actions) and manipulated the visual properties. Our study, together with existing neuroimaging evidence, provides insight to robotics researchers about the visual human qualities of robots that will be designed to interact with humans. It seems that as long as the robot performs the action successfully and is of a sufficiently humanoid design to do so, it will likely be processed in a similar way in the MNS as other people (see also, Gazzola et al., 2007; Oberman et al., 2007; Saygin et al., 2012). Although mu suppression has been linked to the human MNS, it and MNS activity in general do not appear be the right dependent measure for comparing the visual properties of robots with human standards. We suggest that neuroscience studies with mu oscillations as dependent measure might consider using cognitive and affective manipulations to study robot perception or human-robot interaction. It is possible that mu oscillations can inform design issues about the functional properties of robots, rather than visual properties.

Analysis of frontal theta activity on the other hand suggests that theta oscillations can be used as a dependent measure to investigate responses to visual properties of artificial agents, in particular on the interplay between perceptual and memory processes. Our results showed that if the artificial agent is sufficiently humanlike in appearance (Android vs. Robot), it is more likely to facilitate access to semantic representations pertaining to the seen stimuli (here, actions). If the seen agent is rather different from a human in terms of its visual appearance (as in the case of the Robot), it can result in greater processing demands in the observer. Our results suggest that movement kinematics might not be as important as the appearance in influencing the mapping process of the visual input to existing long-term memory representations.

In general, future social, behavioral, and neuroscience research on the perception of robots should distinguish the two dimensions, i.e., visual properties and functional properties (and an interaction of the two) when studying the social acceptability of robots. This would result in a more systematic study of the design issues about social robots and enable determination of the right dependent measures to be used as gold standards in human-robot interaction and robot design. This research in turn will inform social and cognitive neuroscience about the neural basis of human social skills. Our study demonstrates that this interdisciplinary work is useful and fruitful, and progress in this direction will improve our understanding in both fields.

## Conflict of Interest Statement

The authors declare that the research was conducted in the absence of any commercial or financial relationships that could be construed as a potential conflict of interest.
